# Genome-Wide Analysis of the *BBX* Genes in *Platanus* × *acerifolia* and Their Relationship with Flowering and/or Dormancy

**DOI:** 10.3390/ijms24108576

**Published:** 2023-05-11

**Authors:** Gehui Shi, Kangyu Ai, Xu Yan, Zheng Zhou, Fangfang Cai, Manzhu Bao, Jiaqi Zhang

**Affiliations:** 1 National Key Laboratory for Germplasm Innovation & Utilization of Horticultural Crops, College of Horticulture and Forestry Sciences, Huazhong Agricultural University, Wuhan 430070, China; 2 Key Laboratory of Urban Agriculture in Central China, Ministry of Agriculture and Rural Afairs, Wuhan 430070, China; 3 Plant Genomics & Molecular Improvement of Colored Fiber Laboratory, College of Life Sciences and Medicine, Zhejiang Sci-Tech University, Hangzhou 310018, China

**Keywords:** *Platanus* × *acerifolia*, *BBX* gene family, expression patterns, flowering, dormancy

## Abstract

The *B-BOX (BBX)* gene family is widely distributed in animals and plants and is involved in the regulation of their growth and development. In plants, *BBX* genes play important roles in hormone signaling, biotic and abiotic stress, light-regulated photomorphogenesis, flowering, shade response, and pigment accumulation. However, there has been no systematic analysis of the *BBX* family in *Platanus* × *acerifolia*. In this study, we identified 39 *BBX* genes from the *P*. × *acerifolia* genome, and used TBtools, MEGA, MEME, NCBI CCD, PLANTCARE and other tools for gene collinearity analysis, phylogenetic analysis, gene structure, conserved domain analysis, and promoter cis-element analysis, and used the qRT-PCR and transcriptome data for analyzing expression pattern of the *PaBBX* genes. Collinearity analysis indicated segmental duplication was the main driver of the *BBX* family in *P*. × *acerifolia*, and phylogenetic analysis showed that the PaBBX family was divided into five subfamilies: I, II, III, IV and V. Gene structure analysis showed that some *PaBBX* genes contained super-long introns that may regulate their own expression. Moreover, the promoter of *PaBBX* genes contained a significant number of cis-acting elements that are associated with plant growth and development, as well as hormone and stress responses. The qRT-PCR results and transcriptome data indicated that certain *PaBBX* genes exhibited tissue-specific and stage-specific expression patterns, suggesting that these genes may have distinct regulatory roles in *P*. × *acerifolia* growth and development. In addition, some *PaBBX* genes were regularly expressed during the annual growth of *P*. × *acerifolia*, corresponding to different stages of flower transition, dormancy, and bud break, indicating that these genes may be involved in the regulation of flowering and/or dormancy of *P*. × *acerifolia*. This article provided new ideas for the study of dormancy regulation and annual growth patterns in perennial deciduous plants.

## 1. Introduction

The B-BOX (BBX) proteins exist in unicellular and multicellular eukaryotes, named for the B-BOX domain, and belong to the family of zinc finger proteins (ZFP) [1]. The BBX protein family is divided into five subfamilies based on structural features and conserved sequences. Among them, BBX I subfamily (BBX1-6) and BBX II subfamily (BBX7-BBX13) both contain two distinct B-BOX domains and a CONSTANS, CO-like, and TOC1 (CCT) domain; BBX III subfamily (BBX14-17) contains one B-BOX domain and a CCT domain, while BBX IV subfamily (BBX18-BBX25) contains two B-BOX domains without a CCT domain, and BBX V subfamily (BBX26-BBX32) contains only one B-BOX domain without a CCT domain [2].

The B-BOX domain consists of approximately 40 amino acids, which are divided into B-BOX1 and B-BOX2 based on differences in conserved amino acids and zinc-ion binding space [3]. B-BOX is rich in cysteine (Cys), histidine (His), and aspartic (Asp), which are essential for binding to zinc ions, DNA, and protein [4,5,6,7,8]. The CCT domain is found only in the BBX I-III subfamilies, contains 42–43 highly conserved amino acids, and plays an important role in transcriptional regulation [4,9]. In addition to the B-BOX domain and CCT domain, there are the M1-M7 domains, valine–proline (VP) domain, and the nuclear localization signal (NLS) domain in BBX proteins [4]. Among them, the M6 domain influences the functional differentiation of the BBX IV subfamily [10]. The VP domain is a key site for interaction with CONSTITUTIVELY PHOTOMORPHOGENIC 1 (COP1) [11,12], and NLSs domains are involved in the correct localization of BBX proteins in the nucleus [13].

*BBX* genes participate in the signaling cascades of plant growth and development in both direct and indirect ways. On the one hand, BBX proteins can directly bind to downstream genes. On the other hand, they interact with other transcription factors to form heterodimers and jointly regulate the expression of downstream genes. BBX proteins act in association with other transcription factors, such as LONG HYPOCOTYL 5 (HY5) and PHYTOCHROME-INTERACTING FACTORS (PIFs), to enable normal photomorphogenesis in seedlings [14]. BBX proteins regulate flowering through direct and indirect regulation of *FLOWERING LOCUS T (FT)* expression in *Arabidopsis thaliana* [15], *Oryza sativa* [16], *Hordeum vulgare* [17], *Solanum lycopersicum* [18], *Solanum tuberosum* [19], *Chrysanthemum morifolium* [20], and *Beta vulgaris* [21]. In addition, *BBX* genes can regulate the biosynthesis of anthocyanins [22], carotene [23], and chlorophyll [24] and participate in various hormone signaling pathways [25]. Furthermore, *BBX* genes are involved in abiotic stresses, such as light, salt, temperature, and drought, as well as in biotic stresses in *A*. *thaliana* and *Musa nana* [15]. Noteworthy, in perennial tree poplar (*Populus* spp.), *CO (BBX1)-FT* model controls growth cessation and bud set [26]. Therefore, the question arises whether *BBX* genes regulate flowering and/or dormancy in other perennials.

*Platanus* × *acerifolia* is a perennial deciduous tree undergoing a flowering transition in mid to late spring. This is followed by vegetative growth in summer, then enters dormancy in early autumn to late winter, and subpetiolar bud breaks in early spring [27,28]. Previous studies have revealed that two *FT* orthologs, which may act as downstream targets of *BBX* homologous genes, are responsible for regulating flowering and/or dormancy in *P*. × *acerifolia*, respectively [27,29,30]. In this study, we performed the first genome-wide and transcriptome analysis of the *BBX* gene family of *P*. × *acerifolia*. A total of 39 *BBX* genes were identified from the genome, and their phylogenetic relationships, gene structure, conserved domains, and cis-elements of promoter were analyzed. In addition, spatiotemporal expression analysis of *PaBBX* genes was performed via qRT-PCR and transcriptome data. The analyzing results and expression revealed the relationship of *PaBBX* genes with flowering and/or dormancy.

## 2. Results

### 2.1. Identification of BBX Genes in P. × acerifolia

To obtain *P*. × *acerifolia* BBX proteins, the published *Arabidopsis* BBX proteins and B-BOX domain were used to search against the *P*. × *acerifolia* genome via hmmsearch in HMMER. As a result, 39 *BBX* genes were identified in *P*. × *acerifolia*. We named these *BBX* genes according to their closest homologs in *Arabidopsis*. Detailed information, including the gene identifier, chromosome position, protein lengths (the number of amino acids, AA), isoelectric point (pI), and molecular weight (MW) of the corresponding PaBBX proteins, was listed in Table 1. Sequence analysis showed that the *PaBBX* genes varied from 387 bp (*PaBBX30*) to 51,802 bp (*PaBBX10*), while the length of most genes was less than 5000 bp. The lengths of PaBBX proteins ranged from 128 (PaBBX30) to 520 (PaBBX10). The theoretical pI of PaBBX proteins ranged from 4.44 (PaBBX28/29-3) to 9.35 (PaBBX32-1), and the MW varied from 14.90 kDa (PaBBX30) to 58.57 kDa (PaBBX10).

**Figure 1:**
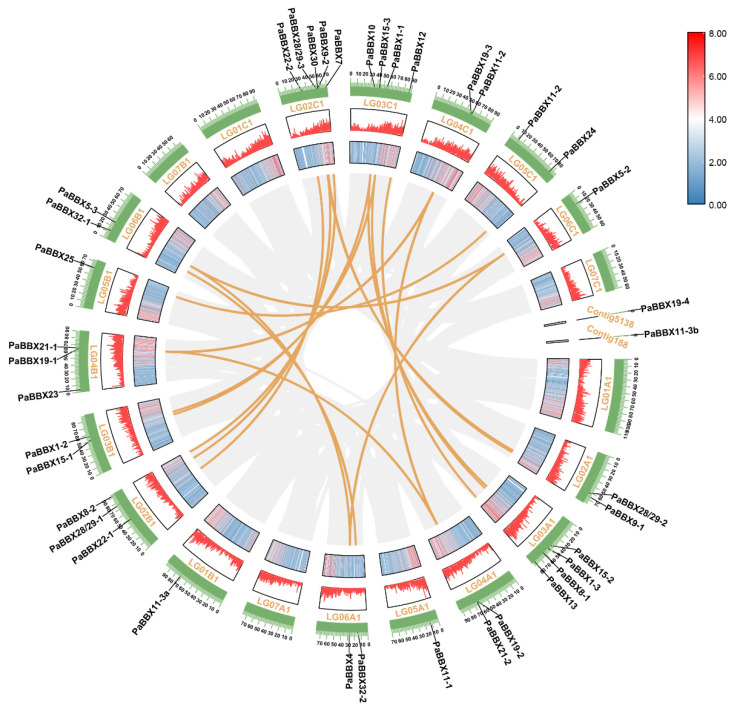
Chromosome distribution and segmental duplication of *PaBBX* genes. The first and second inner rings indicated the density of genes on the chromosome, and the third ring represented the position of the *PaBBX* genes on the chromosome. The distribution and collinearity analysis of *PaBBX* genes were drawn using the Circos program of TBtools software.

There are 42 chromosomes in the *P*. × *acerifolia* genome (Unpublished). The 39 *PaBBX* genes were unevenly distributed among the 16 chromosomes and 2 countings on a set of subchromosomes (Figure 1). Tandem duplications and segmental duplications contributed significantly to the expansion of gene families [31]. So we used TBtools to predict their expansion patterns through gene collinearity analysis. The results showed twelve pairs of duplicated segments (*PaBBX1-1/PaBBX1-2/PaBBX1-3*, *PaBBX4/PaBBX5-1/PaBBX5-2*, *PaBBX7/PaBBX8-2, PaBBX9-1/PaBBX9-2*, *PaBBX12/PaBBX13*, *PaBBX15-1/PaBBX15-2/PaBBX15-3*, *PaBBX19-1/PaBBX19-2/PaBBX19-3*, *PaBBX21-1/PaBBX21-2/PaBBX21-3*, *PaBBX22-1/PaBBX22-2*, *PaBBX24/PaBBX25*, *PaBBX28/29-1/PaBBX28/29-2/PaBBX28/29-3*, and *PaBBX32-1/PaBBX32-2*) identified in the *P*. × *acerifolia* genome (Figure 1). Additionally, we did not find tandem duplication events. This indicates that the expansion of the PaBBX family was mainly due to segment duplication. In addition, the sequences of *PaBBX11-3a* and *PaBBX11-3b* were the same, and the sequences of *PaBBX19-4* were similar to the partial sequence (41-223AA) of *PaBBX19-2*.To investigate the selection pressure in the duplication of the *PaBBX* gene pairs, the non-synonymous (Ka)/synonymous (Ks) values were calculated. The Ka/Ks values of all gene pairs were less than 1.0 (Table S1), indicating that *PaBBX* gene pairs evolved under purifying selection.

### 2.2. Phylogenetic Analysis and Multiple Alignments of BBX Proteins

**Figure 2:**
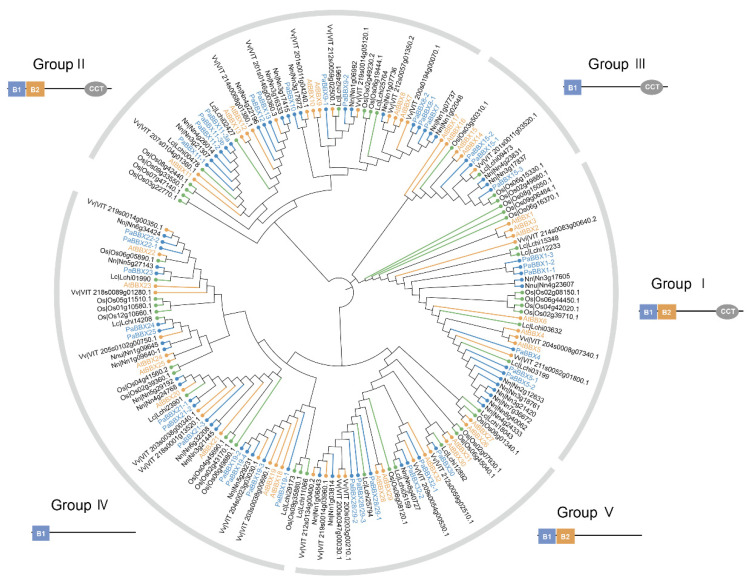
Phylogenetic relationship of BBX transcription factors from *Platanus* × *acerifolia* (Pa), *Arabidopsis thaliana* (At), *Oryza sativa* (Os), *Liriodendron chinense* (Lc), *Nelumbo nucifera* (Nn), and *Vitis vinifera* (Vv). Yellow represented core eudicots, blue represented basal eudicots, and green represented monocots and *Magnoliaceae*. The neighbor-joining (NJ) algorithm-based phylogenetic tree was built using MEGA X software with 1000 bootstrap replicates.

To elucidate the evolutionary and phylogenetic relationships between the PaBBX proteins and other BBX proteins, we created two unrooted phylogenetic trees using MEGA X software with the neighbor-joining (NJ) method. One phylogenetic tree was constructed using the conserved domain sequences of 39 PaBBX proteins together with 32 *A*. *thaliana* BBX proteins, 30 *O*. *sativa* BBX proteins, 18 *Liriodendron chinense* BBX proteins, 32 *Nelumbo nucifera* BBX proteins, and 25 *Vitis vinifera* BBX proteins (Figure 2). Another phylogenetic tree was performed with a full-length sequence of 39 PaBBX proteins (Figure S1). Both phylogenetic trees showed that PaBBX proteins were divided into five groups. BBX I subfamily (PaBBX1-PaBBX5) and BBX V subfamily (PaBBX28/29-PaBBX32) both contained six gene sequences. BBX II subfamily (PaBBX7-PaBBX13) and BBX IV subfamily (PaBBX19-PaBBX25) both contained twelve gene sequences, and BBX III subfamily only contained three gene sequences.

**Figure 3:**
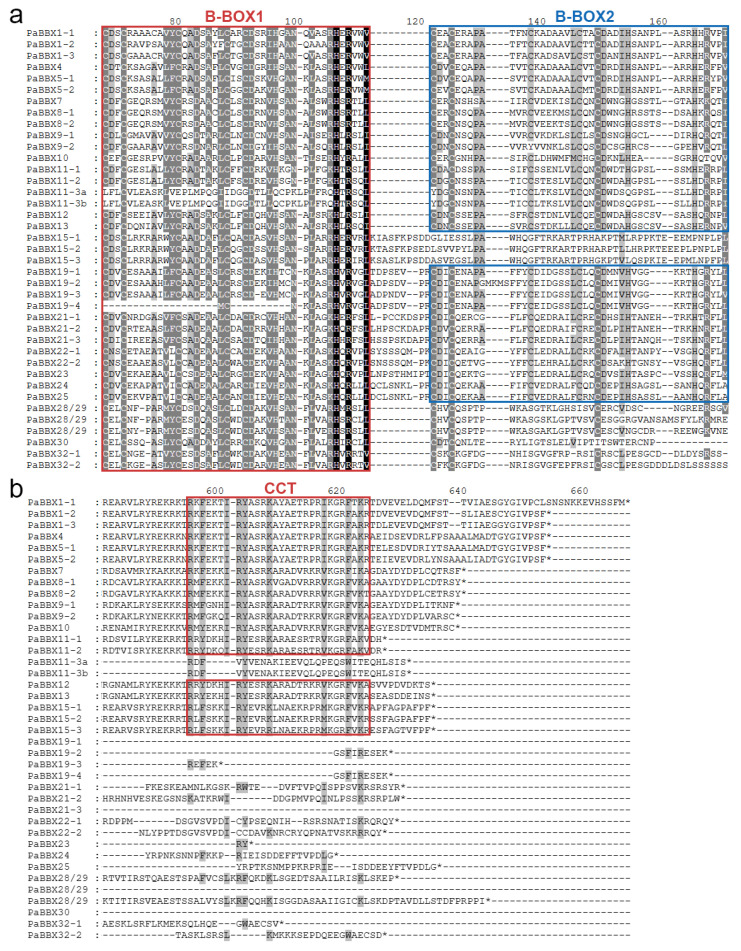
Multiple sequence alignments conserved domain of PaBBX proteins. (**a**) Alignment of the B-BOX domain of PaBBX proteins. (**b**) Alignment of the CCT (CONSTANS, CO-like, and TOC1) domain of PaBBX proteins. Conserved amino acids were marked with red and blue square frames. * indicated the stop codon. Multiple alignments were built using MEGA X software with Clustal W program.

To analyze the conserved domain sequence of PaBBXs protein, we utilized the MEGA X software with Clustal W program to perform multiple sequence alignment of the PaBBXs protein (Figure 3). The results revealed that these proteins contained the highly conserved B-BOX1 with C-D-X-C-X8-CX2-D-X-A-X-L-C-X2-C-D-X3-H-X2-N-X5-H-X-R-X2-L sequence (Figure S2a) and a less conserved B-BOX2 with C-X2-C-X4-A-X2-C6-L-C-X2-C-D-X3-H-X6-H-X-R-X3 sequence (Figure S2b). In addition, the CCT domain was highly conserved (Figure S2c). It is worth noting that PaBBX11-3a/b belonged to BBX II subfamily but lacked B-BOX1 and CCT domains, and PaBBX19-4 belonged to BBX IV subfamily but lacked the B-BOX1 domain. Moreover, there were some amino acid changes in the consensus sequence of B-BOX2. Cys-1 and His-33 of PaBBX11-3a/b were changed to Tyr and Glu, respectively, and Cys-13 of PaBBX9-2 was changed to Tyr (Figure 3a).

In addition to the BOX and CCT domains, the alignments of the conserved sequences of the NLS domains, VP domains, and M1-M7 domains in PaBBX proteins were also presented. The NLS domain was present in BBX I-III subfamilies except for PaBBX11-3a/b because the NLS domain partially overlaps with the CCT domain. Moreover, some BBX IV and V members also had NLS domains (Figure S3a,b). The VP domain appeared only in the BBX I family (Figure S3c). M1-M7 conserved motifs were found in different subfamilies. BBX I subfamily contained M1 and M2 domains (Figure S3d,e); PaBBX7-PaBBX10 of BBX II subfamily contained M3 domains (Figure S3f); BBX III subfamily contained M4 and M5 conserved domains (Figure S3g,h). Except for BBX19, other BBX IV subfamily proteins contained M6 and M7 conserved domains (Figure S3i,j).

### 2.3. Gene Structure and Conserved Motif Analysis

**Figure 4:**
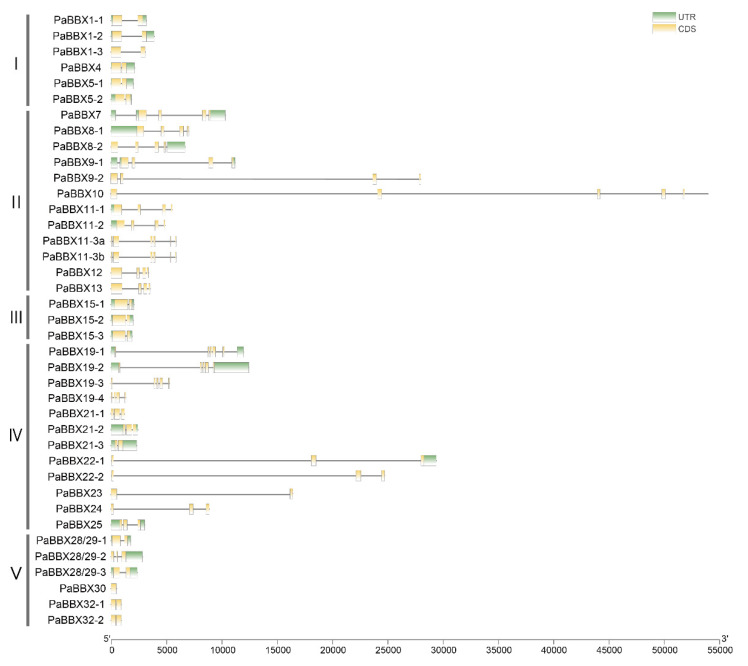
Exon–intron structures of *PaBBX* genes. The exons, introns, and untranslational regions (UTRs) were represented by yellow blocks, thin gray lines, and green blocks, respectively.

To clarify the structural features of the *PaBBX* genes, the exon–intron distribution was analyzed (Figure 4). Except for *PaBBX30*, all other *PaBBX* genes contained introns. Furthermore, the intron lengths varied widely, with the shortest intron being 49 bp of *PaBBX32-1* and the longest being 23,518 bp of the first intron of *PaBBX10*. The intron–exon structures of the *BBX I* and *BBX III* subfamilies genes were relatively similar, with one intron and two exons, while the structure and intron length of the *BBX II* and *IV* subfamilies genes varied widely. Notably, there were five genes with introns over 10,000 bp (Table S2). To verify whether these genes transcribed normally, *PaBBX22-2* and *PaBBX23* were selected as examples. The CDS of *PaBBX22-2* and *PaBBX23* were amplified and proved to be the same as those in the genome. Since introns are a reservoir of cis-elements and also have functional roles [32,33], we predicted the cis-elements on these super-long introns. The results showed that these introns contained a large number of light, temperature, and hormone response elements, tissue-specific expression cis-elements, and biotic and abiotic stress elements (Table S3). Moreover, *PaBBX22-2* and *PaBBX23* also contained AT-rich sequences involved in maximal elicitor-mediated activation (Table S3). This suggested that these super-long introns may have important functions in the regulation of gene expression.

**Figure 5:**
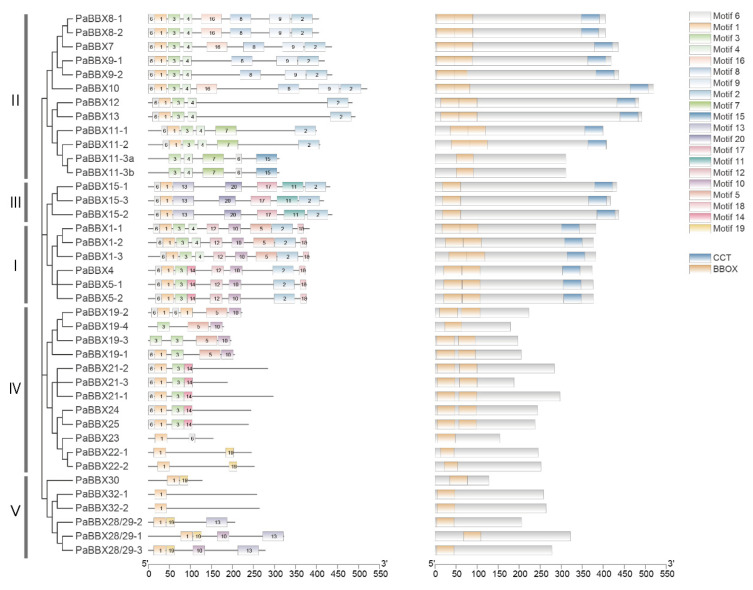
Distribution of the potential motifs and conserved domain of PaBBX proteins. Motifs were named 1–20 and represented by rectangular boxes with different colors. Data were projected via MEME URL.

To better understand the functional divergence of PaBBX proteins, the online MEME tool was used to investigate the conserved motif compositions (Figure 5). As shown in Figure 4, a total of 20 motifs were present in PaBBX proteins. Motifs 1, 3, 4, 6, 14, and 20 were identified as part of the B-BOX domain, while Motif 2 was identified as the CCT domain. In addition to these ubiquitous motifs, some motifs were only presented in a subfamily. For example, Motifs 12 and 18 were presented only in the BBX I subfamily, Motifs 7, 8, 9, 15, and 16 were presented only in the BBX II subfamily, and Motifs 11, 17, and 20 were presented only in the BBX III subfamily. Similar motif compositions were observed in subfamily proteins, while significant divergences were found in different subfamilies, suggesting that PaBBX proteins may have redundancy of functions in the same subfamily and divergence of functions in different subfamilies.

### 2.4. Expression Profiles of PaBBX Genes

**Figure 6:**
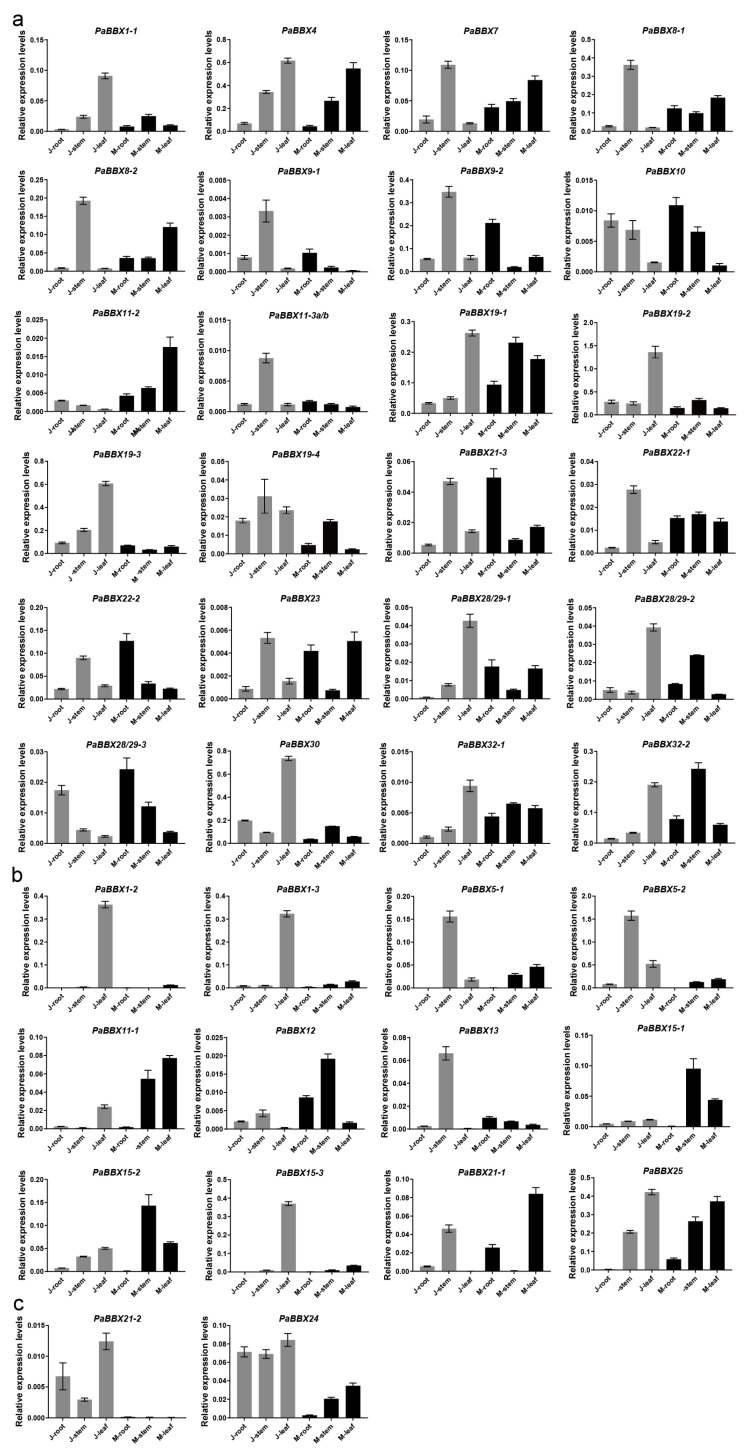
Expression profiles of *PaBBX* genes in various tissues. (**a**) The *PaBBX* genes were ubiquitously expressed in almost all organs tested. (**b**) The *PaBBX* genes were tissue-specific expressed. (**c**) The *PaBBX* genes were stage-specific expressed. J and M represented juvenile and maturely adult trees, respectively. The relative expression level was normalized to the *PaTPI* (*P*. *× acerifolia triose phosphate isomerase*) gene. Data were presented as the mean values ± SD (standard deviation) from three biological replicates.

To predict the potential roles of the *PaBBX* genes, we examined the expression profiles of *PaBBX* genes in various tissues by qRT-PCR, including roots, shoots, and leaves of juvenile and mature adult trees. The sequences of the CDS and untranslated region (UTR) of *PaBBX11-3a* and *PaBBX11-3b* are similar, differing by only a few bases, so it was not feasible to design primers to differentiate between them. Therefore, their expressions were jointly analyzed in this chapter and 2.5. As shown in Figure 6, the expression patterns of *PaBBX* genes could be classified into three groups. The first group was ubiquitously expressed in almost all organs tested, including *PaBBX1-1*, *PaBBX4*, *PaBBX7*, *PaBBX8-1/2*, *PaBBX9-1/2*, *PaBBX10*, *PaBBX11-2*, *PaBBX11-3a/b*, *PaBBX19-1/2/3/4*, *PaBBX21-3*, *PaBBX22-1/2*, *PaBBX23*, *PaBBX28/29-1/2/3*, *PaBBX30*, and *PaBBX32-1/2* (Figure 6a). The second group was tissue-specific expression genes. Among these genes, *PaBBX5-1*, *PaBBX5-2*, *PaBBX15-2*, and *PaBBX25* showed a low level of expression in both juvenile and adult roots. *PaBBX11-1* was hardly detected in roots and juvenile stems, while *PaBBX13* was mainly expressed in juvenile stems. The expression of *PaBBX12* was low in leaves at both stages, and *PaBBX21-1* showed very weak or no expression in juvenile leaves and adult stems, whereas *PaBBX1-2*, *PaBBX1-3*, and *PaBBX15-3* showed significantly higher levels of expression in juvenile leaves. Moreover, *PaBBX15-1* was highly expressed only in the stems and leaves of adult plants (Figure 6b). The third group consisted of genes with stage-specific expressions: *PaBBX21-2* and *PaBBX24.* The expression of these two genes was higher in juveniles, with very weak or no expression in adults (Figure 6c). The different expression patterns suggested that the *PaBBX* genes may participate in different processes of growth and development in *P*. × *acerifolia*.

### 2.5. Transcriptional Profiles of PaBBX Genes during Annual

*CO* promotes *Arabidopsis* flowering under long-day conditions, and *CO*-RNAi poplars limit growth early under short-day conditions [26,34]. To investigate whether *PaBBX* genes were involved in flowering and/or dormancy regulation, such as *CO*, we observed the expression patterns of *PaBBX* genes at the corresponding stages. The expression of *PaBBX* genes throughout the year is presented by two sets of data, and the date from mid-spring to late-summer was based on transcriptomic data, while the date from early autumn to next early spring was obtained from qRT-PCR results.

**Figure 7:**
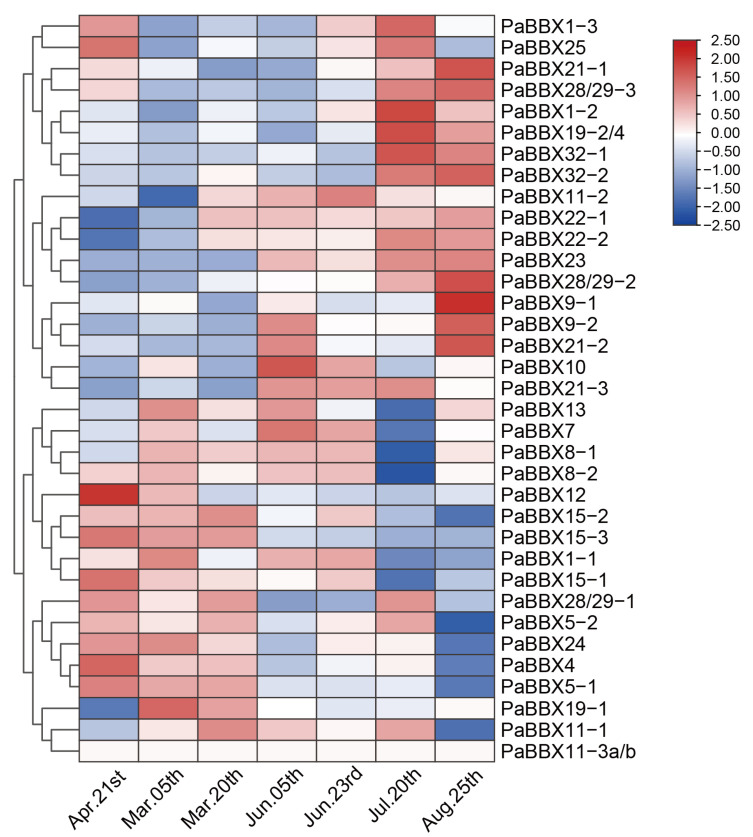
Expression of *PaBBX* genes from mid-spring to mid-summer.

**Figure 8:**
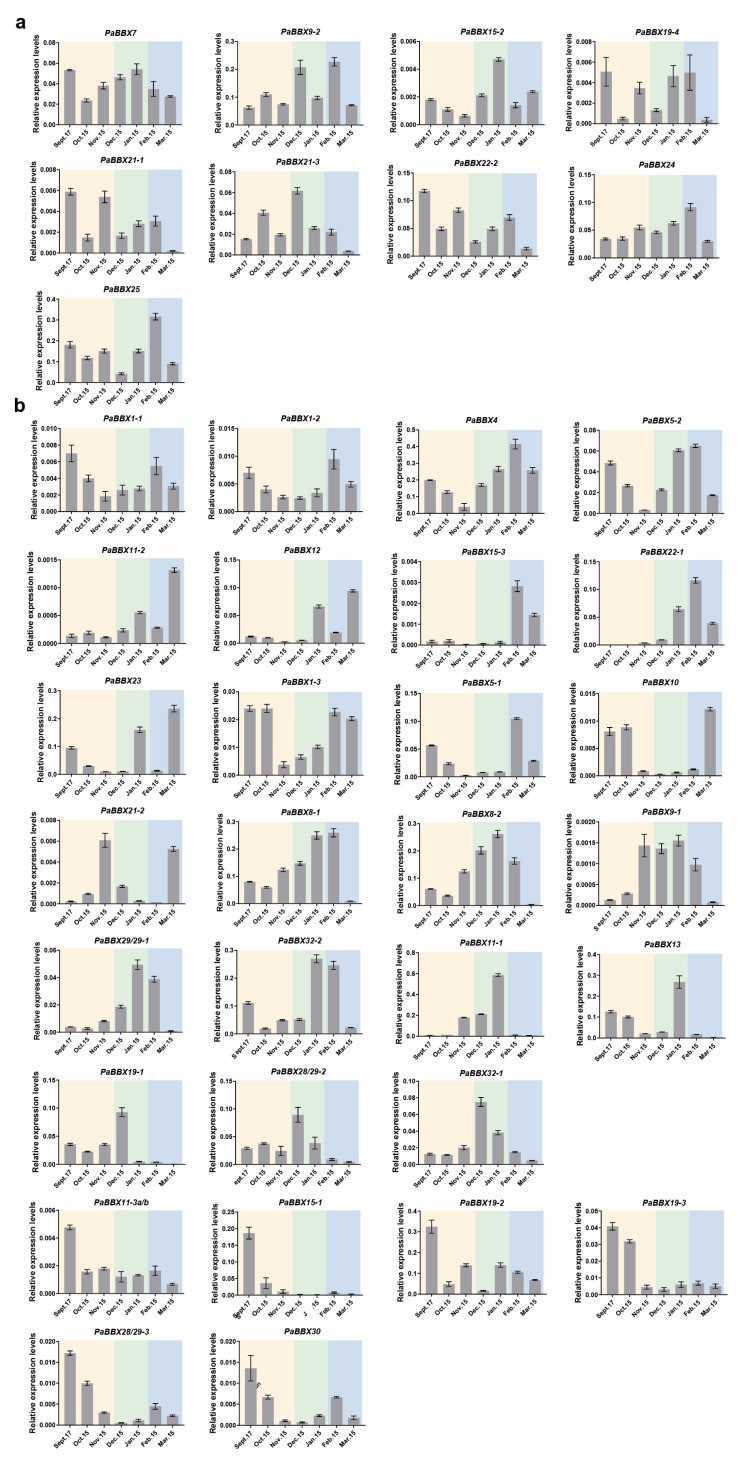
Transcriptional profiles of *PaBBX* genes in dormancy and bud break stage. (**a**) The *PaBBX* genes were irregularly expressed. (**b**) The *PaBBX* genes were regularly expressed. According to the different stages, September to next March was divided into three stages and represented by different colors: yellow (endodormancy), green (dormancy release), and blue (bud break). Data were presented as the mean values ± SD (standard deviation) from three biological replicates.

During the first growth season from mid-spring to late-summer, *PaBBX1-1*, *PaBBX4*, *PaBBX5-1/2*, *PaBBX7*, *PaBBX8-1/2*, *PaBBX11-1*, *PaBBX12*, *PaBBX13*, *PaBBX15-1/2/3*, *PaBBX19-1*, *PaBBX24*, and *PaBBX28/29-1* exhibited high expressions during the flowering transition period (Figure 7). During different periods of dormancy and bud break, eight genes showed irregular expression patterns, including *PaBBX7*, *PaBBX9-2*, *PaBBX15-2*, *PaBBX19-4*, *PaBBX21-1*, *PaBBX21-3*, *PaBBX22-2*, *PaBBX24*, and *PaBBX25* (Figure 8a). On the other hand, some genes showed a regular expression pattern; for example, *PaBBX1-1*, *PaBBX1-2*, *PaBBX4*, and *PaBBX5-2* were highly expressed during endodormancy and bud break (Figure 8b). In addition, several genes showed an opposite pattern of expression. *PaBBX11-2*, *PaBBX12*, *PaBBX15-3*, *PaBBX22-1*, and *PaBBX23* were highly expressed in bud break, while the expressions of *PaBBX11-1 PaBBX13*, *PaBBX19-1, PaBBX28/29-2*, and *PaBBX32-1* were absent during bud break. Moreover, the expressions of *PaBBX1-3*, *PaBBX5 -1*, *PaBBX10*, and *PaBBX21-2* were low in the dormancy release period; however, *PaBBX8-1*, *PaBBX8-2*, *PaBBX9-1*, *PaBBX28/29-1*, and *PaBBX32-2* showed upregulated patterns in dormancy release and the eve of bud break. Moreover, the expression of *PaBBX11-3a/b*, *PaBBX15-1*, *PaBBX19-3*, *PaBBX19-2*, *PaBBX28/29-3* and *PaBBX30* was high in the endodormancy stage (Figure 8b). These expression patterns indicated that some *PaBBX* genes might participate in regulating flowering, dormancy, and bud break of *P*. × *acerifolia*.

### 2.6. Identification of Cis-Elements in the Promoter of PaBBX Genes

**Figure 9:**
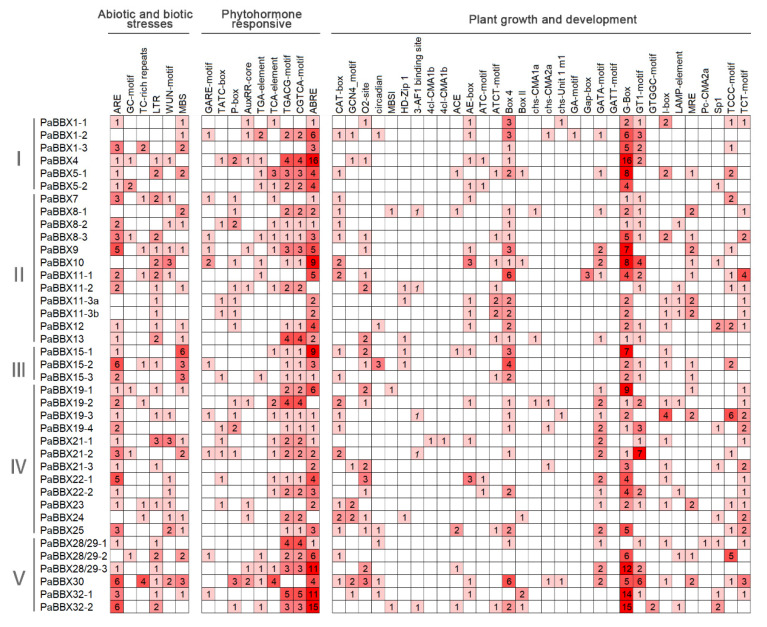
*Cis*-regulatory elements analysis of *PaBBX* genes promoters. Counts were the number of cis-elements.

To explore the potential regulatory mechanism of *PaBBX* genes, the promoter regions (2000 bp upstream sequence from the start codon of the genomic DNA sequence) of the *PaBBX* genes were submitted to the PlantCARE database [35]. Many cis-elements involved in abiotic and biotic stress responses, phytohormone responses, and plant growth and development were identified in addition to the core promoter elements TATA and CAAT boxes (Figure 9 and Table S4). Among the abiotic and biotic stress response elements, anaerobic inducible elements (ARE and GC-motif), defense and stress response elements (TC-rich repeats), low-temperature responsive element (LTR), wound-responsive element (WUN-motif), and drought-inducible elements (MBS) were found in 32, 9, 24, 14, and 21 *PaBBX* genes, respectively. Many elements involved in hormone responsiveness were detected, especially the abscisic acid-inducible element (ABRE), which was abundantly found in the promoters of 36 *PaBBX* genes except *PaBBX11-2*, *PaBBX19-2*, and *PaBBX24*. Meanwhile, gibberellin response elements (GARE-motif, TATC-box, and P-box), auxin response elements (AuxRR-core and TGA-element), salicylic acid response elements (TCA-element), and MeJA response elements (TGACG-motif and CGTCA-motif) were also detected in 25, 18, 17, and 30 *PaBBX* genes, respectively. In addition, 24 different light response elements were detected in the *PaBBX* genes promoters, with the largest number and the widest range of G-box elements. Other elements involved in plant growth and development, such as Meristem expression elements (CAT-box and GCN4_motif), zinc metabolism elements (O2-site), circadian control elements (circadian), flavonoid biosynthetic genes regulation elements (MBSI), and palisade mesophyll cell differentiation elements (HD-Zip 1), were observed in 22, 18, 7, 3, and 6 *PaBBX* genes promoters, respectively. These elements indicate that *PaBBX* genes may be involved in plant growth and development, hormone and defense signaling, and adaptation to environmental changes.

## 3. Discussion

### 3.1. Structural and Evolutionary Analysis of BBX Genes in P. × acerifolia

In this study, we identified 39 *PaBBX* genes from *P.* × *acerifolia*. Phylogenetic analysis revealed that the 39 PaBBX proteins could be classified into five subfamilies, BBX I-V subfamilies (Figure 2 and Figure S1). The same subfamily shared similar exon–intron organization and exon length, except for the BBX IV subfamily (Figure 6). Interestingly, some BBX II and IV genes were much longer than other species due to the super-long intron [36,37,38,39]. There are many cis-elements involved in expression regulation in the super-long introns of *PaBBX* genes (Table S3), suggesting that these introns may be involved in the transcriptional regulation of these genes.

The B-BOX and CCT regions of PaBBX proteins were highly conserved as in other species [36,37,40,41], indicating that their functional differentiation may depend on regions other than BOX and CCT, such as the VP domain, the M6 domain, the PF(V/L)FL motif, and other unknown motifs [4,10,42,43]. In addition, some BBX proteins have lost conserved domains during recent evolutionary events. For example, ZmBBX7 in *Zea mays*, BdBBX11 in *Brachypodium distachyon*, and VviBBX22a in *V*. *vinifera* all lack a B-BOX domain [4,44]. This loss also occurred in *P*. × *acerifolia*, where PaBBX11a/b lacked the B-BOX1 and CCT domains, and PaBBX19-4 lacked the B-BOX1 domain, although other common features were conserved (Figure 5). B-BOX is crucial to the function of *BBX* genes [45], and the loss of the B-BOX domain may affect the function of these genes. Moreover, the conserved amino acids Cys and His of B-BOX2 had been replaced in PaBBX11-3a/b and PaBBX9-2 (Figure 4). This variation is also found in the grapevine and may give rise to novel functions [44].

To explore the evolution of the BBX family, a phylogenetic tree of BBX proteins from *Magnoliales* (*L*. *chinense*), monocots (*O*. *sativa)*, basal eudicots (*N*. *nucifera and P*. × *acerifolia),* and core dicots (*V*. *vinifera* and *A*. *thaliana*) was constructed. Except for some rice proteins, homologous BBX proteins from different species clustered together (Figure 2), suggesting that BBX proteins shared a common ancestor and expanded individually after the divergence of magnolias, dicots, and monocots. Subsequently, the segmental and tandem duplication events were analyzed in order to elucidate the expansion of the *BBX* gene family in *P*. × *acerifolia*. Twelve pairs of duplicated segments, including 30 genes, were found (Figure 1), explaining that segmental duplication is the main driver of the *BBX* family expansion in *P*. × *acerifolia*, as in *Pyrus bretschneideri* [31] and *Fagopyrum Tataricum* [38]. Gene duplication events contribute to the generation of novel functions, and expression profile is one of the characteristics of functional differentiation [46]. *PaBBX* genes generated by segment duplication showed differential spatiotemporal expression (Figure 6, Figure 7 and Figure 8), which may lead to neofunctionalization and subfunctionalization.

### 3.2. Expression Patterns and Potential Functions of PaBBX Genes

The functions of the *BBX* gene family are involved in many processes of plant growth and development, such as seedling photomorphogenesis, flowering, plant hormone signal transduction, pigment accumulation, and abiotic and biotic stresses [15]. However, there have been no studies on the function of *BBX* genes of *P*. × *acerifolia*. Expression level and function are closely related, and gene expression mainly depends on the cis-elements on the promoter [47]. Therefore, we predicted the promoter cis-elements of *PaBBX* genes and performed spatiotemporal expression analysis to anticipate their functions in *P*. × *acerifolia*.

*BBXs* are involved in the vegetative growth of plants. *PtCO1/2* overexpressing poplars were smaller than control plants [48], overexpression of *IbBBX29* resulted in increased biomass of *Ipomoea batatas* leaves [49], and *BBX31* promoted primary root elongation under low-intensity white light in *Arabidopsis* [50]. In *P*. × *acerifolia*, most *PaBBX* genes were detected in all tissues/organs tested but with different expression patterns. *PaBBX1-1/2*, *PaBBX13*, *PaBBX15-3*, and *PaBBX30* were strongly detected in juvenile leaves, implying that these genes may have a similar function in regulating leaf development in juvenile trees. The expressions of *PaBBX5-1/2*, *PaBBX11-3a/b*, and *PaBBX13* were significantly higher in the juvenile stem, suggesting that they may contribute to the internode elongation of stems. *PaBBX28/29-3* and *PaBBX10* were highly expressed in roots at different stages, which may be involved in root growth. In addition, the expression level of *PaBBX21-2* and *PaBBX24* was different between childhood and adulthood and may have different functions in these two periods. The expression patterns of these genes suggested that they may play different regulatory roles in the development of *P*. × *acerifolia*. In addition, different copies of some genes showed almost identical expression patterns (Figure 6), such as *PaBBX1-1* and *PaBBX1-2*, *PaBBX5-1* and *PaBBX5-2*, and *PaBBX8-1*, and *PaBBX8-2*, possibly with redundant functions. However, some other *PaBBX* genes showed differential spatiotemporal expression between copies (Figure 6), suggesting that subfunctionalization and/or neofunctionalization events may also occur between different copies.

Flowering and dormancy have important biological significance for the reproduction and survival of woody plants. Hormones are an important internal factor in regulating flowering and dormancy. GA is the most important hormone in floral transformation, and it works together with ABA antagonism to ensure that plants survive in winter [51,52,53,54,55]. Previous research has shown that the contents of ABA and GAs were tightly linked to the dormancy process of *P*. × *acerifolia* (unpublished). Moreover, almost all *BBX* genes respond to exogenous treatment of phytohormones in different plant species, and some *BBX* genes are involved in the transduction of GA and ABA signaling pathways [25,56,57,58]. Similarly, a large number of hormone response elements were present on the *PaBBX* genes promoter (Figure 9), suggesting that *PaBBX* genes may be involved in these hormonal pathways. In particular, a total of eight *PaBBX* genes contained more than six ABA-response elements, which may be involved in ABA-mediated growth processes such as bud dormancy. Photoperiod and temperature are decisive external environmental factors that regulate flowering and dormancy. Previous evidence suggests that *BBX* genes play an important role in the light signaling pathway [15]. In *P*. × *acerifolia,* each *PaBBX* gene contains numerous light-responsive elements. Among them, G-BOX, the binding site of central light signal regulators such as HY5 and PIFs, is the most abundant and widely distributed [59]. The results suggested that *PaBBX* genes were likely involved in light signaling transcriptional cascades, such as photomorphogenesis and pigment accumulation mediated by *HY5* and *PIFs*, flowering, and shade avoidance. It is noteworthy that 21 *PaBBX* genes have low-temperature responsive elements while chilling in the winter release dormancy [60], indicating that *PaBBX* genes may participate in the dormancy release.

*BBX* genes are known to regulate flowering in herbs [15,61,62,63], and in perennial woody plants populus, *PtCO2* has an effect on poplar growth cessation and bud set [26]. This suggested that the *BBX* genes may be involved in both flowering and dormancy regulation. In our study, a large number of *PaBBX* genes were highly expressed during mid-late spring (Figure 7), indicating that these *PaBBX* genes may be involved in the flowering transition and vegetative growth. In addition, some *PaBBX* genes were regularly expressed, corresponding to different stages of dormancy and bud break. Previous studies have shown that the expression of *PaFT* dramatically increased under low temperatures and short days from early autumn to late winter and was believed to serve to release buds from dormancy [27]. Expression patterns of *PaBBX8-1*, *PaBBX8-2*, *PaBBX9-1*, *PaBBX28/29-1*, and *PaBBX32-2* were similar to these of *PaFT*, suggesting that these genes may act as an upstream regulator of *PaFT* to contribute to the break of dormancy. The expressions of *PaBBX11-2* and *PaBBX15-3*, similar to *EARLY BUD BREAK 1(PtEBB1)*, an important factor in poplar bud break, were not detectable during dormancy but increase rapidly before bud break [64]. Therefore, it is possible that the function of these two genes may be also involved in bud break, such as *PtEBB1*. Moreover, *PaBBX11-3a/b*, *PaBBX15-1*, *PaBBX19-3*, *PaBBX19-2*, and *PaBBX30* displayed high expression levels at the endodormancy phase; these genes may play an important role in dormancy maintenance (Figure 8). Overall, *PaBBX* genes may play vital roles in flowering and/or dormancy in *P*. × *acerifolia* and function at different stages of dormancy. However, the exact function of *PaBBX* genes needs further experimental confirmation.

## 4. Materials and Methods

### 4.1. Plant Materials

For the analysis of gene expression patterns, the adult tree materials were obtained from 40-year-old *P*. *× acerifolia*, field grown at Huazhong Agricultural University, Wuhan, China. The juvenile tree materials were harvested from one-year-old seedlings with 10 leaves. The seedlings were collected from adult plants and grown by sowing them in a growth chamber under LD conditions (16 h:8 h, light/dark, 24 °C).

It has been found that critical period for the flowering transition in *P*. × *acerifolia* occurs during mid to late spring (April to May). The endodormancy period is observed during autumn, which spans from September to November. Dormancy release takes place during early and mid-winter (December and next January). Additionally, the bud break period of *P*. × *acerifolia* occurs during late winter and the next early spring (February and March). For the annual expression pattern analysis, the transcriptome samples were the subpetiolar buds of adult trees from April to August 2021, and the subpetiolar buds of the above adult trees were collected from September 2021 to March 2022.

### 4.2. Identification of BBX Genes from P. × acerifolia

We aligned protein profiles to the verified BBX proteins and BBX domain (PF00643) of *Arabidopsis* via diamond and/or using hmmsearch in HMMER with an E-value of 10^−5^ from *P*. × *acerifolia* genome databases (unpublished). Then, all obtained BBX sequences were confirmed to contain the B-BOX domain by NCBI Conserved Domain Database (CDD) website (https://www.ncbi.nlm.nih.gov/Structure/cdd/wrpsb.cgi accessed on 4 December 2022).

The molecular weight (MW) and isoelectric points (pI) of PaBBX proteins were calculated on the ExPASy website (https://web.expasy.org/protparam/ accessed on 6 December 2022).

The distribution and collinearity analysis of *PaBBX* genes on chromosomes were drawn using the Circos program of TBtools software [65]. The nonsynonymous (Ka) and synonymous (Ks) substitution rates of the gene duplication pairs were estimated via TBtools.

### 4.3. Phylogenetic Analysis and Protein Sequence Alignments

The BBX protein sequences of *Arabidopsis* were downloaded from the Arabidopsis Information Resource (TAIR) database (http://www.arabidopsis.org accessed on 6 December 2022), and the sequences of *Liriodendron chinense*, *Oryza sativa*, *Nelumbo nucifera*, *Vitis vinifera* were obtained from the Phytozome database (https://phytozome-next.jgi.doe.gov/ accessed on 6 December 2022) [66]. Protein sequence alignments of BBX proteins of *P*. × *acerifolia, Arabidopsis, L*. *chinense*, *O*. *sativa*, *N*. *nucifera*, and *V*. *vinifera* were conducted using the Clustal W program of MEGA X software. Phylogenetic tree of BBX in six species and only in *P*. × *acerifolia* based on the alignments and constructed by the neighbor-joining (NJ) method using the Poisson model and 1000 replicates of bootstrap value. Then, the tree was uploaded to iTOL (https://itol.embl.de/ accessed on 14 December 2022) to beautify [67]. Sequence prediction of NLS, VP domain, and M1-M7 domains were performed according to previous articles [4,43]. Sequence logos for generating conserved domains were generated using the online WebLogo (http://weblogo.berkeley.edu/logo.cgi accessed on 8 December 2022).

### 4.4. Gene Structure and Conserved Motif Analysis

The exons, introns, and untranslational region (UTR) of the BBX genes were identified according to the genome annotation of *P*. × *acerifolia*. The conserved domains were predicted via the CDD database, and conserved motifs were identified via Multiple Expectation Maximization for Motif Elicitation (MEME) online database (http://memesuite.org/tools/meme accessed on 27 December 2022). The maximum motif number in MEME was set as 20, and the motif length was set as 6–50 amino acids. The gene structure and conserved motif of *PaBBX* genes were drawn using TBtools software.

### 4.5. Putative Cis-Element Analysis of PaBBX Genes Promoters and Intron

The 2000bp region upstream from the start codon of the *PaBBX* gene was extracted from the *P*. × *acerifolia* genome as a promoter, and introns of *PaBBXs* about 10 kbp were also extracted from the *P*. × *acerifolia* genome. Then, the sequences of promoters and introns were uploaded to the Plantcare website (http://bioinformatics.psb.ugent.be/webtools/plantcare/html/ accessed on 9 December 2022) to predict putative *cis*-acting element [35].

### 4.6. Cloning PaBBX22-2 and PaBBX23

Based on the genome data of *P*. × *acerifolia* (unpublished), primers were designed by Primer 5. The mixed-buds cDNA was used as the template. PCR amplifications were performed in a 10 μL volume reaction containing 1.0 μL template, 4.5 μL 2 × Hieff PCR Master Mix (Yeason, Shanghai, China), 0.5 μL forward and reverse primers (10 μL mol/μL), and 3.5 μL water. The PCR reaction program is 95 °C for 3 min; 95 °C for 30 s, 56 °C for 30 s, 72 °C for 1 min (40 cycles); 72 °C for 10 min. The product of PCR amplification was cloned to PMD18-T vector (TaKaRa, Otsu, Japan) for sequencing. The primers used are shown in Table S5.

### 4.7. Analysis Expression Pattern of PaBBX Genes

For the expression analysis of *PaBBX* genes, RNA from the plant material in 4.1 was extracted via CTAB according to a previously reported protocol [68], and complementary DNA (cDNA) was synthesized using PrimeScriptTM RT reagent Kit with gDNA Eraser (TaKaRa, Otsu, Japan). Quantitative Real-time PCR (qRT-PCR) was performed using cDNA as a template and using SYBR Premix Ex TaqTM (TaKaRa, Otsu, Japan) in an Applied Biosystems 7500 Fast Real-Time PCR System (Applied Biosystems, Waltham, MA, USA) and following the manufacturer’s instructions. The qRT-PCR amplifications were performed in a 10 μL volume reaction containing 1.0 μL template, 5.0 μL 2 × SYBR Green Master Mix, 0.2 μL forward and reverse primers (10 μL mol/μL), and 3.6 μL water. The qRT-PCR reaction program is 95 °C for 30 s, 95 °C for 5 s, and 60 °C for 34 s (40 cycles). Primers for qRT-PCR were designed using the Primer3Plus website (https://www.primer3plus.com/ accessed on 13 December 2022). The *PaTPI* (*P*. × *acerifolia triose phosphate isomerase*) was used as reference genes to normalize all data. The expression levels of a relative gene were detected using the method of 2^−∆∆CT^. Data were presented as the mean values ± SD (standard deviation) from three biological replicates. In addition, expression from April to August 2021 is based on unpublished transcriptome data. *PaBBX19-3* and *PaBBX30* did not obtain expression data because they did not annotate. The histogram was drawn using GraphPad Prism 9, and the hierarchical clustering heatmap was performed by TBtools software. The primers used are shown in Table S5.

## 5. Conclusions

In conclusion, like the *BBX* family in *Arabidopsis*, the *BBX* family in *P*. × *acerifolia* was divided into five subfamilies, BBX I-V subfamilies, with a total of 39 *BBX* genes. The same subfamily has similar gene structures and conserved motifs, but different subfamilies have different gene structures and conserved motifs. In addition, some genes contain super-long introns that may regulate their expression. The expansion of the *P*. × *acerifolia BBX* family was mainly caused by segmental duplication and had undergone purifying selection. The promoters of the *PaBBX* genes contained many elements related to plant growth and development as well as hormone and stress response. Expression pattern analysis showed that the expression of some BBX genes was closely related to the process of flowering and/or dormancy, which may be involved in the regulation of flowering and/or dormancy of *P*. × *acerifolia*. This study is the first comprehensive analysis of the *BBX* gene family in *P*. × *acerifolia* on a genome-wide scale, reveals the relationship of *PaBBX* genes with flowering and/or dormancy, provides new ideas for the regulation of flowering and/or dormancy in perennials, and enriches the genetic stock of *P*. × *acerifolia* for molecular breeding.

## Figures and Tables

**Table 1:** *BBX* genes identified in *P*. × *acerifolia*.

Gene Name	Gene Identifier	Chromosomes Position	AA	MW (kDa)	pI
*PaBBX1-1*	PaC3G126980	53,321,272–53,324,171	383	42.81	6.04
*PaBBX1-2*	PaB3G200600	57,602,006–57,605,538	377	41.98	5.68
*PaBBX1-3*	PaA3G246120	51,710,999–51,713,790	383	42.22	5.93
*PaBBX4*	PaA6G276070	27,421,637–27,423,559	374	41.07	5.33
*PaBBX5-1*	PaB6G308930	27,080,769–27,082,589	376	41.39	5.38
*PaBBX5-2*	PaC6G408130	17,664,745–17,666,673	377	41.29	5.56
*PaBBX7*	PaC2G374890	68,135,516–68,144,895	436	47.57	5.25
*PaBBX8-1*	PaA3G242790	61,010,653–61,017,031	405	44.36	5.59
*PaBBX8-2*	PaB2G072670	91,892,447–91,898,521	405	44.44	5.01
*PaBBX9-1*	PaA2G259070	66,033,109–66,043,294	419	45.96	5.69
*PaBBX9-2*	PaC2G370070	56,914,445–56,942,510	439	48.10	5.47
*PaBBX10*	PaC3G123980	36,237,251–36,289,053	520	58.57	5.88
*PaBBX11-1*	PaA5G347500	12,207,675–12,212,906	400	45.51	5.93
*PaBBX11-2*	PaC5G216800	12,060,240–12,064,960	409	45.22	5.16
*PaBBX11-3a*	PaB1G042960	71,884,034–71,889,372	311	35.02	5.04
*PaBBX11-3b*	PaUnG429150-RA	43,527–48,865	311	35.02	5.04
*PaBBX12*	PaC3G139600	87,665,264–87,668,302	485	53.53	6.12
*PaBBX13*	PaA3G234590	79,284,256–79,287,445	492	54.23	6.8
*PaBBX15-1*	PaB3G199040	51,735,509–51,737,376	432	48.20	4.97
*PaBBX15-2*	PaA3G247750	46,315,091–46,315,360	437	48.34	4.95
*PaBBX15-3*	PaC3G125090	42,957,754–42,959,478	417	46.43	5.11
*PaBBX19-1*	PaB4G179910	64,824,309–64,835,187	204	23.14	5.34
*PaBBX19-2*	PaA4G084960	64,411,679–64,420,184	223	24.95	6.19
*PaBBX19-3*	NA	58,602,036–58,606,617	197	21.99	6.38
*PaBBX19-4*	PaUnG529740-RA	2505–3689	179	20.12	6.22
*PaBBX21-1*	PaB4G180130	65,601,829–65,602,921	297	32.81	6.36
*PaBBX21-2*	PaA4G084640	65,243,318–65,245,509	284	31.62	6.2
*PaBBX21-3*	PaC4G152800	59,354,451–59,356,546	188	20.76	6.28
*PaBBX22-1*	PaB2G060740	48,920,313–48,948,818	299	32.77	5.33
*PaBBX22-2*	PaC2G363350	33,447,397–33,470,603	297	32.56	5.84
*PaBBX23*	PaB4G165570	3,297,234–3,310,372	206	22.34	6.43
*PaBBX24*	PaC5G232000	76,674,127–76,682,184	244	26.85	4.9
*PaBBX25*	PaB5G340870	61,197,235–61,199,987	238	26.37	4.87
*PaBBX28/29-1*	PaB2G067580	76,340,288–76,341,894	322	35.13	4.61
*PaBBX28/29-2*	PaA2G260070	61,929,831–61,932,394	206	22.36	8.63
*PaBBX28/29-3*	PaC2G369370	55,193,130–55,195,276	278	29.96	4.44
*PaBBX30*	NA	56,910,595–56,910,981	130	14.90	8.06
*PaBBX32-1*	PaB6G307570	13,158,377–13,159,201	258	28.71	9.35
*PaBBX32-2*	PaA6G274390	14,746,991–14,747,818	264	29.09	8.76

AA: Amino Acid, pI: isoelectric point, MW: molecular weight.

## Data Availability

The data that support the findings of this study are available from the corresponding author upon reasonable request.

## Supplementary Materials

The following supporting information can be downloaded at: https://www.mdpi.com/article/10.3390/ijms24108576/s1.
