# Supplementary material for: Genome-Wide Analysis of the BBX Genes in Platanus × acerifolia and Their Relationship with Flowering and/or Dormancy
Source: Int J Mol Sci. 2023 May 11;24(10):8576. doi: 10.3390/ijms24108576 (PMC10218012; doi:10.3390/ijms24108576)
Supplement: Supplementary file 1 [file ijms-24-08576-s001.zip › ijms-2326191-supplementary figures.pdf]

**Supplementary Materials**

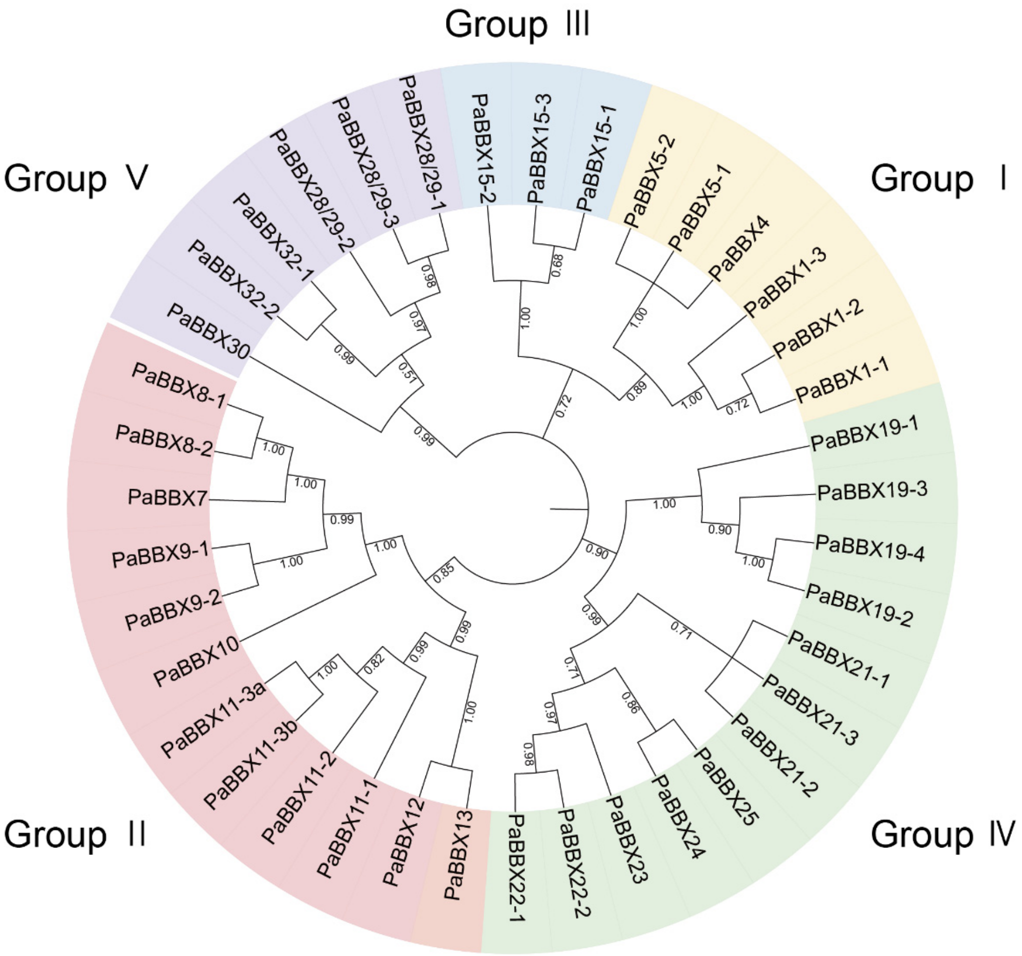


**Figure S1.** Phylogenetic tree of PaBBX proteins. Yellow, pink, blue, green, and purple represent groups I, II, III, IV, V, respectively.

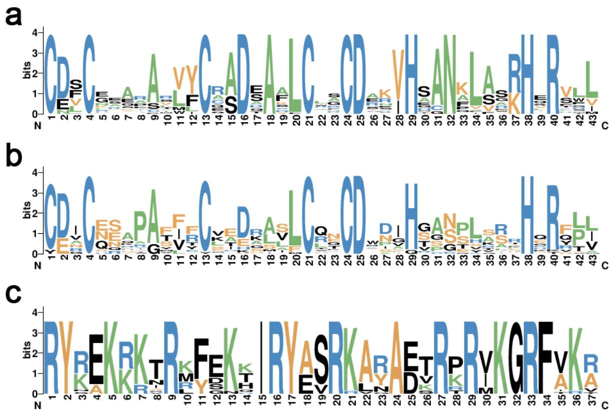


**Figure S2.** Logos of the conserved domains in the PaBBX proteins. (**a**), (**b**) and (**c**) are the protein alignment of the B-BOX1, B-BOX2 and CCT domain, respectively.

The x-axis shows the conserved sequences of the domain. The y-axis represents the conservation rate of each amino acid.

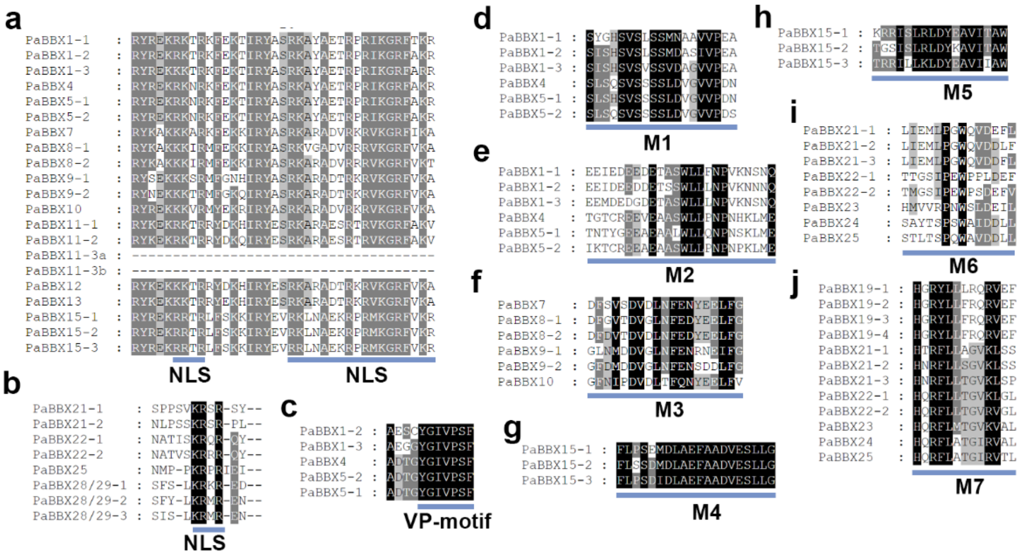


**Figure S3.** Multiple sequence alignments conserved domain of PaBBX proteins. (**a**) and (**b**). Alignment of the nuclear localization signals (NLSs) domain of PaBBX proteins. (**c**). Alignment of the VALINE-PROLINE (VP) domain of PaBBX proteins. (**d**)-(**j**). Alignment of the M1-M7 domains of PaBBX proteins. Conserved amino acids were marked with blue lines.
